# Can We Produce Heparin/Heparan Sulfate Biomimetics Using “Mother-Nature” as the Gold Standard?

**DOI:** 10.3390/molecules20034254

**Published:** 2015-03-05

**Authors:** Brooke L. Farrugia, Megan S. Lord, James Melrose, John M. Whitelock

**Affiliations:** 1 Graduate School of Biomedical Engineering, University of New South Wales, Sydney, NSW 2052, Australia; E-Mails: m.lord@unsw.edu.au (M.S.L.); james.melrose@sydney.edu.au (J.M.); j.whitelock@unsw.edu.au (J.M.W.); 2 The Raymond Purves Research Labs, Institute of Bone and Joint Research, Kolling Institute of Medical Research, University of Sydney, The Royal North Shore Hospital of Sydney, St. Leonards, NSW 2065, Australia

**Keywords:** heparan sulfate, heparin, low molecular weight heparin, glycosaminoglycans, proteoglycans

## Abstract

Heparan sulfate (HS) and heparin are glycosaminoglycans (GAGs) that are heterogeneous in nature, not only due to differing disaccharide combinations, but also their sulfate modifications. HS is well known for its interactions with various growth factors and cytokines; and heparin for its clinical use as an anticoagulant. Due to their potential use in tissue regeneration; and the recent adverse events due to contamination of heparin; there is an increased surge to produce these GAGs on a commercial scale. The production of HS from natural sources is limited so strategies are being explored to be biomimetically produced via chemical; chemoenzymatic synthesis methods and through the recombinant expression of proteoglycans. This review details the most recent advances in the field of HS/heparin synthesis for the production of low molecular weight heparin (LMWH) and as a tool further our understanding of the interactions that occur between GAGs and growth factors and cytokines involved in tissue development and repair.

## 1. Introduction

Glycosaminoglycans (GAGs) are long polysaccharides that are made up of repeating disaccharide units. Within the GAG family there are four main subgroups: (1) hyaluronic acid or hyaluronan (HA), (2) keratan sulfate (KS), (3) chondroitin/dermatan sulfate (CS/DS) and 4) heparan sulfate (HS)/heparin. These subfamilies differ in monosaccharide components, glycosyl linkages, and position and degree of saccharide functionalization. With the exception of HA, GAGs are found covalently attached to a protein core and together are known as proteoglycans. HS and heparin consist of the disaccharide units α(1→4) linked d-glucosamine (GlcN) and (1→4) linked uronic acid which are either α-linkage L-iduronic acid (IdoA) or β-linkage d-glucuronic acid (GlcA). They are heterogeneous in nature not only due to differing disaccharide combinations but also the sulfate modifications. Possible modifications include 2-*O*-sulfation of uronic acid, and *N*-sulfation, *N*-acetylation, 3-*O*- and 6-*O*-sulfation of glucosamine, as such there are potentially 2,916 possible HS pentasaccharide sequences [1]. Almost every cell produces GAGs which are incorporated into proteoglycans in a cell-associated glycocalyx that populates the extracellular matrix (ECM) to define tissue form and function [2,3]. HS has been shown to modulate cell growth and development by regulating growth factors such as the fibroblast growth factor (FGF) family, platelet derived growth factor (PDGF), and vascular endothelial cell growth factor (VEGF) [4,5]. Much progress has been made in recent years in the elucidation of the contribution that GAGs make to tissue development and ECM remodeling in health and disease [6,7].

HS is synthesized by most cells in the body for the sequestration and signaling of many growth factors and cytokines as well as modulating the activities of the protein core to which it is attached and is involved in cell signaling events including proliferation, migration and differentiation at each stage of tissue development and repair [8]. Thus the ability to synthesize HS on a large scale or of a defined saccharide sequence has the ability to more broadly impact advances in medicine and treatment of disease through materials, regenerative medicine and tissue engineering strategies that utilize HS structures specific for the repair or replacement of specific tissues. 

While HS is produced by most cells, heparin is produced exclusively by mast cells. Much research has been focused on the isolation and structural characterization of heparin as it is used clinically as an anticoagulant. Recent adverse events that resulted in approximately 100 deaths in the US caused by an uncontrolled anaphylactoid response [9] due to doping with oversulfated chondroitin sulfate [10] and prompted the recall of heparin sodium from one supplier. This has encouraged various research groups to attempt to recombinantly express heparin in the laboratory as a future alternative to the animal-sourced heparin currently used in the clinic. This approach utilizes recombinant expression of proteoglycans in mammalian cells [11] that can post-translationally modify the protein core with HS/heparin structures, however optimization of experimental conditions is still required before we can recombinantly expressed controlled HS/heparin structures and translate them to clinical application.

## 2. Heparan Sulfate/Heparin Biosynthesis

**Figure 1:**
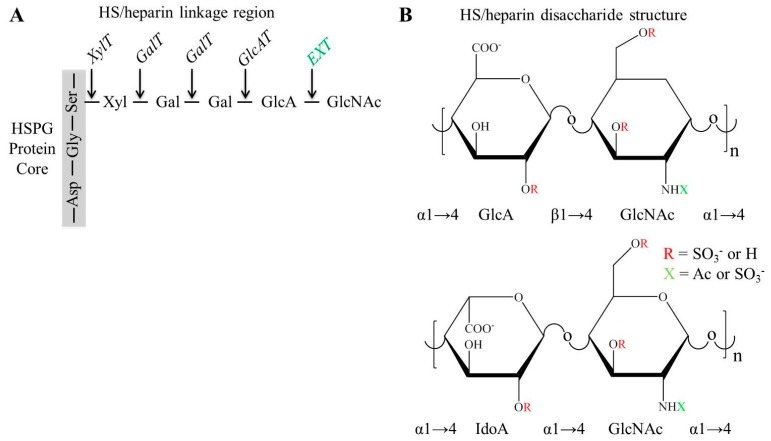
(**A**) Linkage region of GAGs illustrating the amino acid sequence within the PG protein core (serine (Ser), glycine (Gly) and aspartic acid (Asp)) required for covalent attachment of initial saccharide of the linkage region, xylose, where addition of GlcNAc via EXT is where GAG becomes a HS/heparin and diverges from CS synthesis route. (**B**) Comparison of disaccharide unit structures of HS and heparin and the functional groups present within tissues.

HS and heparin are synthesized as long polysaccharide chains that are covalently bonded to the protein core of proteoglycans via an oxygen moiety on a serine residue that is part of a GAG attachment sequence. They are never synthesized as free chains and can decorate the protein core of cell surface proteoglycans such as syndecans and glypicans in addition to the extracellular proteoglycans including perlecan, type XVIII collagen and agrin. In addition to the varied chain lengths, sulfate modifications to the HS chain provide areas of charge that form the basis of the interactions to clusters of basic amino acids on many different types of proteins. Heparin carries significantly more sulfate and charge than HS and is the GAG that specifically decorates the proteoglycan serglycin present in the granules of mast cells where it binds to and controls the activities of many proteases. The synthesis of a HS/heparin chain is fast taking approximately a minute [12,13] yet is also dynamic and complex requiring the concerted action of at least 22 enzymes, some of which have many isoforms that are used to produce cell and tissue specific forms [14]. Most of these enzymes contain a transmembrane region that enables them to be intercalated into the Golgi membranes with those responsible for HS and heparin biosynthesis localized to the more proximal regions in contrast to those enzymes that are integral to CS biosynthesis, which are localized to distal Golgi membranous stacks [15]. The first important step in the synthesis of a HS/heparin chain is the formation of the linkage tetrasaccharide which is initiated by the coupling of a xylose to the serine via the action of xylosyltransferase (XylT) 1 or 2, followed by the addition of two galactose molecules in sequence by galactosyltransferase (GalT) 1 and 2, respectively. The tetrasaccharide structure, which is also common to CS chains, is completed by the addition of GlcA by glucuronic acid transferase (GlcAT) 1. It is at this point in GAG biosynthesis that the HS/heparin biosynthetic pathway diverges from the CS and dermatan sulfate (DS) pathway by the addition of an *N*-acetylglucosamine (GlcNAc) instead of a *N*-acetyl galactosamine (GalNAc) (Figure 1A), though the exact mechanisms responsible in controlling this switch between the major GAG types are largely unknown.

The enzymes, of which there are three known isoforms that are responsible for the addition of the GlcNAc to the non-reducing end of the linkage tetrasaccharide are known as the EXTL family of glycosyltransferases. These enzymes may also be involved in regulating the ratio of HS to CS and controlling the length of the HS chain by forming complexes with each other and the substrate sugars. The polymerization or elongation of the HS chains involves a complex being formed between EXT1 and EXT2 that sequentially add a GlcA followed by a GlcNAc moiety and this gives rise to the characteristic alternating copolymer of these two monosaccharides seen in all HS and heparin chains. The sulfation of HS/heparin is started when the acetyl group on some GlcNAc residues is removed and sulfated by one of the four isoforms of the *N*-deactylase *N*-sulfotransferase (NDST) enzymes. NDST2 has been shown to have specificity for modifying the GAG chains decorating serglycin making it a critical enzyme in the process of synthesizing mast cell heparin [16]. After this sulfation step, further modification of the HS/heparin chains happens in close proximity to these regions leading to relatively highly sulfated domains or S domains. Most forms of HS contain approximately 30% of their sequence decorated with sulfate leaving 70% as undecorated GlcA-GlcNAc sequences [17] whereas heparin contains long stretches of highly sulfated disaccharides. Once the *N*-sulfate modification has taken place, some of the GlcA residues in these regions become epimerized to iduronic acid (IdoA) followed by the vast majority being sulfated at the C2 position. The glucuronyl C5-epimerase and hexuronyl 2 sulfotransferase enzymes both have only a single isomer and co-localize in the Golgi [18], and they have been shown to interact with each other as well as the interaction between C5-epimerase and 6-*O*-sulfotransferase [19]. Sulfate groups can be added to the C6 position of either GlcNAc or GlcNS by one of three isoforms of the 6-*O*-sulfotransferase enzymes. These enzymes can act on both GlcNAc and GlcNS but have been shown to have a preference for modifying regions where there is a higher proportion of GlcNS flanked by 2 sulfate modification residues, which supports the synthesis of longer S domains [20]. It is interesting to note that the rarest sulfate modification present on HS/heparin is carried out by members of the 3-*O*-sulfotransferase family of enzymes, which has the greatest number of isoforms of the seven known isotypes, only three have a transmembrane region suggesting that these might not localize to the Golgi membranes [16]. These enzymes are capable of adding a sulfate to the 3 position of a GlcNS giving rise to the most highly sulfated regions in the S domains (Figure 1B). The most well studied of these is the anti-thrombin III (AT-III) binding pentasaccharide sequence, GlcNS-GlcA-GlcNS3S-IdoA2S-6SGlcNS [21,22]. It is this region of HS/heparin that contain the small amounts of the triply sulfated disaccharides that are also very important for the binding of many heparin binding growth factors and proteins and may be expressed transiently in developing tissues to finely tune tissue structure but the primary role of these groups on HS/heparin remain elusive [23]. The most predominant form for the sulfotransferase 3-*O*-ST1 was knocked down in mice without any effects on coagulation hemostasis suggesting that this form of the enzyme does not modify HS/heparin to bind to AT-III [24]. Instead these animals exhibited a pro-inflammatory phenotype due to endothelial cell dysfunction [25].

## 3. Chemical Synthesis of HS 

Chemical synthesis of HS/heparin involves either building oligosaccharide sequences from disaccharides or chemical modification of polysaccharides to incorporate sulfate groups. The synthesis of HS/heparin oligosaccharides is an inherently difficult process due to the number and type of reactions required, and the low yields commonly achieved. There have been a number of reviews published on the synthetic synthesis of HS/heparin as well as GAG structures in general [26,27,28]. The most recent literature has focused on the innate areas of difficulty within this field including the development of glycosidic acceptors and donors, synthesis methods to increase yield, particularly of oligosaccharides of longer disaccharide length, and libraries of discrete oligosaccharide structures.

### 3.1. Oligosaccharide Synthesis

Modular synthesis of HS/heparin disaccharides involves combining monosaccharides to form disaccharides and then combining these to form oligosaccharides. The formation of disaccharides from monosaccharides, including glycosyl acceptors and donors, through the α(1→4) GlcNAc-GlcA disaccharide precursors is challenging due to the formation of both α- and β-linkages which are not only difficult to separate post-synthesis but most often results in the formation of anomers, decreasing the overall product yield. One of the first reports of HS/heparin oligosaccharide modular synthesis with complete stereoselectivity was achieved via conformational locking of the uronic acid glycosyl acceptor at the reducing end, resulting in exclusive formation α-linked disaccharides [29]. Furthermore, complete stereo-selectivity has recently been demonstrated by Zulueta *et al.* [30] through use of selected protecting groups on a d-glycosaminyl donor. Functionalization of disaccharides for HS/heparin synthesis can occur at C3, C6 or N of GlcNAc and C2 of uronic acids for sulfation. This requires the introduction of specific protecting groups during the formation of the disaccharides, which are then removed to allow sulfo functionalization. Protecting groups are required to be added and removed with a relative amount of ease and not be affected by intermittent reactions. Protecting groups that consist of a levulinoyl ester (Lev), 9-fluorenylmethoxycarbonyl (Fmoc), a *tert*-butyldiphenylsilyl ether (TBDPS) and an allyl ether (All) in combination with monosaccharides are able to produce all the structural elements of HS [31]. Additionally, 2,2,2-trichloroethoxycarbonyl (Troc) has been used for oligosaccharide synthesis on a polymer support [32]. 

HS/heparin chemical synthesis, in general, entails building oligosaccharides from repeating disaccharide units resulting in a homogeneous structure. Synthesis of structures that more closely resemble heterogeneous HS/heparin structures found *in vivo* include methods to produce oligosaccharides of irregular disaccharide structure [33]. Continual research into synthesizing irregular HS oligosaccharide structures has resulted in three hexasaccharides with similar backbone structures differing in functional group placement [34]. The three hexasaccharides were used to investigate binding affinity of 22 different heparin binding proteins helping to understand how differences in oligosaccharide structure influence biological interactions. Further progress in the synthesis of HS oligosaccharides includes the development of new glucuronic acid donors via use of the protecting group of uronic acid to eliminate the need for post glycosylation oxidation [35]. Glucuronic acid donors have previously been reported to behave poorly however they can give higher yields depending on the position and protecting groups utilized. 

Oligosaccharide synthesis is presently difficult to achieve with research focused on reducing the number of reactions required as well as methods to purify the product following each synthesis step. The number of reaction steps has been successfully reduced using a number of approaches including mixture [36] and one-pot [37] synthesis methods. Where the synthesis of particular disaccharides followed by combining matching of donor and acceptor pairs resulted in desired α(1→4) stereoselectivity producing a library of 12 hexasaccharides containing varying backbones with 2-*O*-sulfated uronic acids (IdoA2S and GlcA2S) [38]. The use of fluro tags has been also been employed in HS modular synthesis to separate out the products [39]. Subsequently, an increase in product yield as well as an increase in the rate of sulfation modification resulted as the reactions were able to proceed with higher efficiency. Another technique that has been explored to increase efficiency has been to carry out the synthesis on a solid support rather than in solution [40,41], as well as for the development of iduronic acid and idose donors [42]. 

Synthesizing HS/heparin oligosaccharides of specific structures is desirable to determine structure-function relationships for specific GAG sequences found within the heterogeneous population of chains that are naturally produced by all cell types. Discovery of a specific structure within the heparin polysaccharide chain that is responsible for the anticoagulant activity, GlcNS-GlcA-GlcNS3S-IdoA2S-6SGlcNS has led to the desire to replicate this structure using synthetic chemistry. Fondaparinux, sold under the trade name Arixtra^®^, is a pentasaccharide factor Xa inhibitor developed by Sanofi-Aventi (Paris, France). The original synthesis comprised of more than 50 steps, though more recently was able to be synthesized in only 36 steps, using three protecting groups, however, the yield is very low at 0.017% [43]. Early research into the development of HS mimetics demonstrated through the synthesis of an oligosaccharide structure that mimicked specific NS and NA-NA/NS structures was shown to inhibit the interaction between heparain and γ-interferon [44]. More recent studies in determining structure-function relationships of HS/heparin include synthesis of 12 tetra-saccharides, containing differing disaccharide combinations and sulfate functionality to investigate specific disaccharide structure [45]. Key features of the synthesis methods developed include the use of Lev for the protection of hydroxyl groups requiring sulfation, Fmoc for the protection of C-4' that would then become a glycosyl acceptor and a standard set of reactions that can be used in combination to produce any HS disaccharide structure desired, as well as showing selective inhibition of a protein involved in Alzheimer’s disease based on oligosaccharide structure, for development of a therapeutic. More recently, increasing oligosaccharide length up to dodecasaccharides [46] demonstrated that the biological activity of these oligosaccharides with the same sulfation pattern varied based on length. Synthesis of oligosaccharides ranging from 7 to 12 units in length consisting of α(1→4) GlcN-IdoA disaccharide units, and sulfated to result in populations either containing 2-*O*-sulfate of IodA, or in addition *N*-sulfate of glucosamine [47]. Furthermore, synthesis of a dodecasaccharide with a 45% overall yield has been reported for the investigation of CD4 linked HS as a human immunodeficiency virus inhibitor [48]. Increased oligosaccharide length and sulfation was shown to suppress fibroblast growth factor-2 (FGF-2) and vascular endothelial growth factor-165 (VEGF_165_) induced endothelial cell responses. The [GlcNS6S-IdoA2S]_n_ structure has been synthesized and used to investigate the role of sulfation patterns of GlcNS6S in the regulation of angiogenic cytokine activity [49]. Further work investigating the affinity between the HS disaccharide structures and growth factors has been shown using a library of 48 disaccharides [50], where 24 structures contained the GlcN-GlcA sequence and the other 24 GlcN-IdoA. The disaccharides were generated in 3–7 steps and with an overall yield of between 24%–95%. An investigation of the affinity between these structures and FGF-1 found common features of disaccharides that bound to the growth factor were the GlcN *N*-sulfonate and the IdoA 2-*O*-sulfonate moieties. 

### 3.2. Backbone Modification

Chemical synthesis of GAG mimetics via backbone modification, rather than constructing GAGs using disaccharide building blocks, requires the modification of polysaccharide structures to incorporate functional groups, predominantly sulfo groups. While this approach for synthesis of GAG mimetics is not as difficult as oligosaccharide synthesis there are disadvantages including heterogeneous populations of products and harsh reaction conditions required for the sulfating agents. Backbone modification of polysaccharides is achieved by incorporation of sulfate groups, where O–H or N–H bonds are substituted with an O–S or N–S bond depending on the sulfating agent, the experimental conditions, and the disaccharide units of the polysaccharide being modified. Incorporation of sulfate groups into the polysaccharide backbone has been explored using various reagents including sulfuric acid [51], oleum [52], pyridine-sulfur trioxide [53], sulfating reagent N(SO_3_Na)_3_ [54], and chlorosulfonic acid [55]. One of the first reports of the sulfation of polysaccharides utilized sulfuric acid [51] which resulted in a heterogeneous reaction mixture and depolymerisation of the polysaccharides. It was also shown that increasing the reaction time increased the sulfur content however the downside was a further decrease in molecular weight of the sulfated product. 

#### 3.2.1. Cellulose and Starch

Cellulose and starch are polysaccharides comprised of repeating d-glucose units that differ by type of glycosidic linkage and oligosaccharide length. Their sulfated derivatives have been explored for many applications as their properties are similar to HS/heparin, including anticoagulant activities and growth factor interactions [56]. Sulfation of cellulose acetate or trimethylsilyl cellulose has been explored using sulfating agents, including sulfamic acid and sulfur trioxide or chlorosulfonic acid, with varying degrees of sulfation [57]. It was shown that the degree, as well as the position of sulfation, influenced the anticoagulant properties of the materials. In general there was an increase in anticoagulant activity with increasing degree of sulfation as determined by both thrombin clotting time (TT) as well as partial thromboplastin time (PTT). Further work by Groth *et al.* [56,58] explored the mitogenic activity of the sulfated cellulose materials and demonstrated an increase in fibroblast proliferation in the presence of sulfated materials through interactions with FGF-2. These materials were shown to bind to bone morphogenic protein-2 (BMP-2) to a larger extent than heparin [56] which resulted in an increased osteogenic activity and increased amount of alkaline phosphatase (ALP) of the myoblast cell line, C2C12. An alternative method of sulfation has been explored for both cellulose and starch by using the sulfating agent formed by reacting sodium bisulfate with sodium nitrite forming the sulfating agent N(SO_3_Na_3_)_3_ [59,60,61]. An advantage of this sulfating agent is that it is not hydrolytic, which causes degradation of the polysaccharide backbone, and the reaction can be carried out in an aqueous environment, alleviating the need to use organic solvents. This method has been successfully used to sulfate hydroxymethyl cellulose at the C2 and C6 positions and the resultant materials were shown to increased clotting time, determined by the activated partial thromboplastin time (APTT), with increasing degree of sulfation (from 0.5 to 1.67) [60]. Additionally, APTT was depended on the molecular weight of the sulfated material. Similar results were reported with sulfated carboxymethyl starch [59] and carboxymethyl cellulose [61], though, these materials did not affect the PTT or TT. These results suggest that incorporation of sulfate groups affected the intrinsic clotting or surface clotting pathways rather than the extrinsic pathway.

#### 3.2.2. Alginate 

Alginate, a naturally occurring polysaccharide derived from algae, with repeating disaccharide units of d-mannuronic acid and L-guluronate with β(1→4) glycosidic linkages. Sulfation of alginate using chlorosulfonic acid has been show to produce a material with anticoagulant properties [62]. Heparin-binding growth factor interactions with sulfated alginate were found to be an order of magnitude higher affinity for platelet derived growth factor-ββ (PDGF-ββ), vascular endothelial growth factor (VEGF) and stromal cell derived factor-1 compared with heparin interactions with these same growth factors while FGF-1 and -2 interactions with the sulfated alginate were of a similar affinity to interactions with heparin [63]. *In vivo* release of FGF-2 from loaded sulfated alginate hydrogels implanted into rats was shown to increase vascularization into the porous implants [63], with further studies investigating the release of angiogenic factors, VEGF, PDGF-ββ and transforming growth factor-β1 (TGF-β1) [64]. Additionally, sequential release of these GFs from alginate scaffolds resulted in the formation of blood vessels following implantation into a rat model. Sustained release of the growth factor hepatocyte growth factor (HGF) from an injectable alginate solution that incorporated sulfated alginate was demonstrated by Ruvinov *et al.* [65] as well as a significant increase in blood vessels in mice straight after acute myocardial infarction. Human mesenchymal stem cells (hMSC) encapsulated in alginate sulfate, binding TGF-β, demonstrated the ability to drive the hMSC down a chondrogenic pathway [66]. Mhanna *et al.* [67] demonstrated that the culture of chondrocytes in sulfated alginate hydrogels with a varying percentage of sulfated materials, were able to proliferate and produce a cartilage-like extra cellular matrix (ECM) to support the chondrocytes. HGF bound to GAGs on the surface of myeloma cells was able to be displaced by sulfated alginates [68] indicating that the HGF had a higher affinity for the sulfated alginates than natural HS on the cell surface.

#### 3.2.3. Hyaluronan

Hyaluronan (HA) is the only unsulfated GAG and is uniquely synthesized in the plasma membrane as compared to the Golgi where all of the other GAGs are synthesized. HA is made up of repeating disaccharide units of GlcA and GlcNAc, with β(1→4) and β(1→3) glycoside linkages. The structure of HA is similar to CS, in the absence of sulfate groups where CS contains *N*-acetylgalactosamine (GalNAc), therefore the majority of the literature on HA backbone modification through incorporation of sulfate groups aims to mimic and is compared to CS as opposed to HS. An early report of HA modification via sulfur trioxide [69] demonstrated increasing Anti-IIa activity with increasing amount of sulfation. Modification of HA though sulfation has been shown with low and high levels of sulfation incorporated using SO_3_-pyridine and SO_3_-formaide respectively [70]. BMP-4 binding was limited to low sulfated-HA and HS and increased with high sulfated-HA. 

#### 3.2.4. K5 Polysaccharide 

The polysaccharide K5 is produced via recombinant expression in *E. coli*, and contains the heparin precursor structure β(1→4)GlcA-α(1→4)GlcNAc [71]. Due to the similarities with heparin, K5 has been widely used in HS/heparin mimetics. An early report of sulfated K5 demonstrated the antithrombotic potential of these materials due to their ability to inhibit Factor Xa [72]. *N*-, *O*-, and *N*,*O*-sulfation of K5 polysaccharide has been achieved using pyridine-sulfur trioxide [73] with varying degree of sulfation, where material interaction with FGF-2 and associated receptors was demonstrated as well as the materials potential antiangiogenic activity. Similar materials have also been shown to have possible use in anti-AIDS therapies due to interactions with HIV-1 transactivating factor (Tat) [74]. The ability to produce HS/heparin mimetics termed “neoheparin” in gram quantities has been demonstrated by Lindahl *et al.* [75]. Modification of K5 to produce “neoheparin” included conversion of GlcA to Idoa via C-5 epimerisation, and sulfation incorporation was achieved via per-*O*-sulfation, with an overall production yield of ~60% through the six step functionalization process. 

#### 3.2.5. Chitosan

Chitosan is a polysaccharide produced from the deacetylation of chitin, derived from a variety of sources including crustaceans and algae. Chitosan consists of repeating GlcN and GlcNAc disaccharide units linked by β(1→4) glycosidic bonds, where the ratio of glucosamine to *N*-acetylglucoasmine is dependent on degree of decacetylation. One of the first reports comparing anticoagulant activity of sulfated chitosan showed APTT results of 331–379 U/mg for *O*-sulfated *N*-acetylchitosan, and 190–297 U/mg for *N*,*O*-sulfated chitosan as compared to 174 U/mg for heparin [52]. While sulfated chitosan was initially explored as an anticoagulant, more recent literature has been directed towards its application in growth factor delivery. The ability of sulfated chitosan to enhance osteoblast differentiation in the presence of BMP-2 was demonstrated by Zhou *et al.* [76], where it was shown that the position of the sulfate, whether C2, C6 or C2,6, altered the degree of BMP-2 associated activity through ALP activity and osteoblast mineralization. Encapsulation of BMP-2 into nanoparticles made from sulfated chitosan enhanced human umbilical vein endothelial cell tubule formation on MatriGel, though this was dependent on concentration. Evaluation of bone mineralization and blood vessel formation in a critical sized defect in a rabbit model [77] revealed that sulfated chitosan significantly increased both bone and vessel formation at 8 weeks following implantation while BMP-2 loaded with sulfated chitosan further enhanced bone mineralization and vessel formation. Similar sulfated chitosan nanoparticles were encapsulated in hydrogels with BMP-2 where implanted samples were shown to increase bone formation as compared to growth factor only loaded hydrogels [78], demonstrating the use of chitosan with sulfate motifs as a growth factor delivery system. 

Chitosan containing arginine functionalization was further modified to contain sulfate motifs using chlorosulfonic acid [79]. The ability to differentiate human fetal chondroblasts to a cartilage or bone phenotype was demonstrated following exposure to chitosan-arginine materials or materials containing sulfate motifs, respectively. Exposure of embryonic stem cells to various sulfated chitosans, with 2-*N*, 6-*O*, 3,6-*O* or 6-*O* functionalization, were shown to express the neuron specific marker βIII-tubulin, where expression varied with both sulfate position and degree of sulfation [80]. Indicating that sulfate decorated materials are able to induce stem cell differentiation. 

## 4. Enzymatic Synthesis of HS 

The chemoenzymatic approach utilizes enzymes to synthesize and/or modify polysaccharides to produce GAGs. Within the literature there are a number of recent review articles that have focused on this method for GAG synthesis [81,82,83]. Glycosyltransferases (GTases) add monosaccharides from UDP-sugar donors onto an acceptor. Sulfotransferases (STases) transfer sulfo groups from the donor, 3'-phosphoadenosine-5' (PAPS) to the GAG chain and the control of epimerases that convert GlcA into IdoA. The conversion of GlcA to IdoA via C5-epimerase is a reversible reaction *in vitro* [84]. There are several *N*- and *O*-sulfotransferases (OSTs) that add a sulfo group to the glucosamine (GlcN) residue in HS. There are multiple isozymes with distinct substrate specificities [85], and all, with the exception of some HS polymerases, have been recombinantly expressed in *E. coli* [86]. Similar to the biosynthesis of HS/heparin, the sequence in which enzymes act is based on the state of the preexisting saccharide structure. OSTs selectivity is based on the sulfation state of the substrate, where 2-OST requires *N*-sulfation of the adjacent GlcN residue and epimerization of GlcA to IdoA requires the presence of *N*-sulfo groups. Strategies to improve chemoenzymatic synthesis include the use of flurotags to speed up the purification process, a relatively new method in chemoenzymatic synthesis [87]. Cai *et al.* [88] recently reported the use of fluorous tag *tert*-butyl dicarbonate as a reducing-end tag in the synthesis of HS tetra- and hexasaccharides. The tag was shown not to interfere with the enzymatic processes, and for future work the disaccharide structure present at the reducing end is a potential starting point for the chemoenzymatic synthesis of Arixtra^®^. Similarly to chemical synthesis current research in this area includes the use of a solid support [89] as well as one pot synthesis [90]. 

### 4.1. UDP-Sugar Synthesis 

A limitation of chemoenzymatic synthesis of GAGs is the cost and availability of UDP-sugars. UDP-sugars are available in both natural and unnatural forms, where UDP-IdoA is not naturally available and its chemical synthesis is important. Synthesis of UDP-IdoA allows the addition of this saccharide straight into the GAG chain rather than via epimerization [91]. In addition to UDP-IdoA, UDP-GlcA and UDP-HexUA have been synthesized for enzymatic incorporation into HS/heparin oligosaccharides. More recently a number of analogues that are precursors to UDP-sugars, based on UDP-GlcNAc and UDP-GalNAc have been developed [92] and are being used to determine structures required for recognition by *N*-acetylglucosamine-1-phosphate uridyltransferases to produce the UDP-sugars and increase the number of these saccharides available for GAG synthesis. 

### 4.2. Oligosaccharide Synthesis

Formation of GAGs from UDP-sugars requires participation of particular enzymes that include the family of *Pasturella multocida* heparosan synthases. These synthases are present as two different isoforms, PmHS1 and PmHS2, while both will produce heparosan, their catalytic phenotypes are different. To investigate the structure-function relationship attributed to the different isoforms a series of chimeric enzymes were developed that incorporated different segments of the two isoforms into one enzyme [93]. The sequences within the enzymes that are responsible for donor binding and acceptor usage were reported, furthering our understanding of the relationship between enzymatic structure and activity. Synthesis of size defined *N*-sulfo-saccharides up to 21 units in length have been used to investigate the effect of structure on anticoagulant activity [94]. Anti-Xa activity was shown with the shorter oligosaccharides, five saccharide units in length, which implies the length of the oligosaccharide does not influence activity. In contrast, anti-IIa activity was dependent on longer oligosaccharides as shown with a 21-mer. Methods to decrease the number of synthesis steps to produce similar oligosaccharides [95] was achieved via one-pot synthesis method where synthesis time was decreased from 14 to 2 days for production of a dodecasaccharide. Synthesis of ultra-low molecular weight heparins, five to 10 saccharide units in length, have been shown to have comparable pharmacokinetic properties to Arixtra^®^ [96]. Additionally, synthesis was achieved in 10–12 steps, compared to ~50 steps using synthetic chemistry and an overall yield of between 37%–45%. Further work has confirmed the need for IdoA2S, not GlcA2S, for AT binding to the oligosaccharide to occur [97]. More recently, the homogeneous production of low molecular weight heparin [98], through controlling C5-epimerase, has achieved the IdoA2S-GlcNS repeating disaccharide structure.

HS is well known for its interactions with growth factors, particularly the FGF family. The binding of synthesized heptasaccharides to FGF-2 found that binding to the growth factor was due to structural differences rather than charge [99], demonstrating the importance of IdoUA in the binding of FGF-2. The signaling of FGFs occurs through formation of a ternary complex of FGF-HS-FGFR, though the structure of this complex is well known whether it is symmetric or asymmetric remains unclear. A series of well-defined oligosaccharides, 40 saccharide units in length, were synthesized and demonstrated that the FGF-HS-FGFR complex is symmetric [100]. 

## 5. Recombinant Expression of HS/Heparin Proteoglycans

Proteoglycans are comprised of protein cores that are post-translationally modified with one or more GAG chains. Proteoglycan domains, including perlecan, agrin, syndecans and glypicans, have been recombinantly expressed in mammalian cells as a method to produce HS. GAG synthesis is not a DNA/RNA template driven process but instead requires multiple Golgi and endoplasmic reticulum localized enzymes to be expressed including those involved in GAG chain elongation, epimerization of GlcA and modification by the addition of sulfates along the chain. To date mammalian cell lines that have been explored for the recombinant production of HS/heparin include the Chinese hamster ovary (CHO) and human embryonic kidney (HEK-293 and HEK-293T) cells as they express the enzymes involved in GAG synthesis. A challenge that remains for the production of HS and heparin is the synthesis of these chains with the desired chain length and sulfation pattern as it is currently not known how to precisely control the structure of the GAG chains produced by recombinant means. Frequently the recombinant production of proteoglycan domains results in the synthesis of CS and this is thought to be due to the residence time of the protein core in the Golgi [101]. Additionally, a proportion of the protein core produced may be secreted without post-translational modification or with short GAG chains. This is likely due to the production of the protein core exceeding the maximum velocity of the enzymes involved in GAG synthesis leading to an increase in the heterogeneous nature of the GAG chains produced. One promising approach that is able to add some control over the HS/heparin chain structures produced is transfection of CHO-S cells with the enzymes involved in GAG synthesis and modification and these cells are capable of producing HS. These cells produce two of the three HS 6-*O*-sulfotransferases (HS6st), two of the four *N*-deacetylase/*N*-sulfotrasnferases (NDST) and none of the 3-*O*-sulfotransferases (HS3st) [102]. Transfection of the NDST2 and HS3st1 enzymes into CHO-S cells successfully produced HS with a higher ratio of NS to OS than without transfection of these enzymes into the CHO cells [103,104]. Additionally, CHO-S cells transfected with Golgi targeted HS3st1 resulted in the production of HS chains containing the AT-III binding site as well as more disaccharides containing 2-*O*-, 6-*O*- and *N*-sulfo group [105]. Similarly, transfection of murine mastocytoma cells with HS3st1 resulted in increased expression of 3-*O*-sulfo groups in the HS disaccharides and processed anti-coagulant activity [106].

### 5.1. Perlecan

Perlecan is the major HS proteoglycan of basement membranes and is also present in connective tissues and certain blood cells. Perlecan has a protein core of ~460 kDa while its GAG chains can range in size from 10 to over 200 kDa, depending on the cell source. Of the five domains, the *N*-terminal domain I is the smallest being approximately 20 kDa and contains three GAG attachment sites, serines 65, 71 and 76, that are most commonly decorated with HS. Amino acid sequences upstream of these serine residues are important in determining the type of GAG chains that decorate these serine residues [107], however expression of domain I alone most often results in decoration with both HS and CS. Domain I of perlecan expressed in HEK-293 cells was decorated with both HS and CS [108,109,110] The HS chains contained unsulfated HS disaccharides as well as *N*-aceteylated, monosulfated disaccharides and *N*-sulfated disaccharides. This recombinant perlecan domain I HS was found to bind FGF-2 to the same extent as immunopurified endothelial cell derived perlecan [110] and augment BMP-2 activity [111]. Similarly human perlecan domain I expressed in 293 EBNA cells was decorated with both HS and CS and was found to bind FGF-2 [112] as well as VEGF_165_ [113]. Mouse perlecan domain I expressed in HEK-293 cells produced two populations, one decorated entirely with HS and the other decorated with both HS and CS/DS with average chain lengths of 8–10 kDa [114]. Expression of mouse perlecan domain I in CHO-K1 cells resulted in the production of approximately equal amount of HS and CS/DS chains decorating the protein core with an average size of 12 kDa [115]. The expression of human perlecan domain I together with enhanced green fluorescent protein in HEK-293 cells reduced overall expression levels, but resulted in the GAG produced being entirely HS [108]. 

The C-terminal domain V of perlecan is approximately 90 kDa and consists of laminin-type G modules as well as epidermal growth factor-link modules as well as one GAG attachment site at serine 3511 [116]. This domain of mouse perlecan has been expressed in HEK-293, EBNA-293 and COS-7 cells and found to contain two proteoglycan populations, one containing HS and the other containing CS [116,117,118] while a population without GAG substitution has also been reported [116,117]. Expression of human perlecan domain V in HEK-293 cells is also produced as two populations, one with HS and the other with CS [119] while a shorter form of domain V without the BM40 leader sequence expressed in HEK-293 cells results in a protein form of human perlecan domain V, also termed endorepellin [120]. Proteoglycan forms of perlecan domain V have been used to demonstrate that the HS and CS chains are anti-adhesive for smooth muscle cells, while endothelial cells can bind to domain V irrespective of the presence of the GAG chains [121].

### 5.2. Agrin

Agrin is an extracellular matrix proteoglycan involved in the formation of the neuromuscular junction with a protein core of approximately 220 kDa and GAG chains of up to 180 kDa [122]. The HS chains that decorate agrin play a role in the inhibition of neurite outgrowth [123]. Regions of agrin containing serine-glycine repeats have been expressed in HEK-293 cells. Two of the nine peptides expressed were found to be decorated with GAGs, one peptide exclusively decorated with HS while the other was predominantly decorated with CS and a minor amount of HS [124]. 

### 5.3. Syndecans

The syndecans are a family of four cell-surface HS proteoglycans that are important for cell motility as well as growth factor and ECM molecule binding. The ectodomain of syndecan-1 expressed in CHO cells was decorated with HS including the disaccharides 2-*O*-sulfated iduronic acid-*N*-sulfated, 6-sulfated glucosamine and 2-*O*-sulfated iduronic acid-*N*-sulfated glucosamine and found to bind FGF-2 [125]. In contrast, spliced forms of syndecan-1 expressed in Madine-Darby canine kidney cells resulted in a mixed population decorated with both HS and CS [126]. Similarly, a recombinant fusion proteoglycan containing both syndecan-4 and FGF1 expressed in CHO cells was also a mixed population decorated with both HS and CS [127].

### 5.4. Glypicans

The glypicans are a family of six cell-surface HS proteoglycans, and like the syndecans, interact with growth factors and ECM molecules via their HS chain. The glypicans contain a cysteine-rich globular domain, a GAG attachment region and a glycosylphosphatidylinositol membrane anchor. The human glypican-1 ectodomain expressed in HEK-293 cells was decorated with HS [128]. Rat glypican-1 expressed in COS cells was predominantly decorated with HS, with some CS while the globular domain was found to be essential in obtaining a high level of HS substitution [129].

### 5.5. Serglycin

Serglycin is an intracellular proteoglycan produced by many different cell types including endothelial cells, platelets, neutrophils and mast cells, with the latter being the only cell type to decorate the protein core of serglycin with heparin [130]. The other cell types decorate the eight GAG attachment sites in the protein core with HS, CS and DS [131,132,133]. Serglycin is important for the intracellular storage of proteases, cytokines and growth factors in secretory granules, such as in white blood cells and platelets. Serglycin produced by mast cells is thought to be the major source of commercial heparin isolated from porcine intestinal mucosa or bovine lungs. Serglycin has been expressed in rat pancreatic acinar cells [134] and while the specific GAGs that decorated the protein core were not explored, it was revealed that the presence of GAGs was essential for trafficking of the serglycin to the granules. In the future the recombinant expression of serglycin under conditions that promote HS/heparin biosynthesis may be explored. 

## 6. Conclusions 

With close to 1000 possible HS/heparin pentasaccharide sequences the range of HS/heparin sequences present *in vivo* that control tissue development, repair and regeneration is vast. The field of biomimetic HS/heparin via chemical and chemoenzymatic synthesis and recombinant expression approaches has only produced a few of the possible structures that will be required to advance medical technologies and treatment regimes. New glycosyl acceptors and donors, methods to achieve desired stereoselectivity to new UDP-sugar donors and transfection of enzymes involved in GAG synthesis into mammalian cells are all recent advances in the different methods utilized to produce biomimetic HS and heparin. Improvements to chemical synthesis techniques have led to increased oligosaccharide production yields and the production of a LMWH via enzymatic means that has shown to have similar pharmacokinetics to Arixtra^®^. However, caution must be taken and vigorous clinical testing carried out to ensure the mechanism by which these synthetic mimics function *in vivo* truly replicates heparin to ensure the adverse, and potentially lethal, effects are once again not seen. These advances have led to potential new antithrombotic and anticoagulant therapeutics and the ability to produce diverse range of oligosaccharide structures has helped to understand the structure function relationship occurring between GAGs and growth factors, cytokines that regulate cellular differentiation and proliferation during tissue development. 
